# Identification and Evaluation of Methodologies to Assess the Quality of Mobile Health Apps in High-, Low-, and Middle-Income Countries: Rapid Review

**DOI:** 10.2196/28384

**Published:** 2021-10-12

**Authors:** Fionn Woulfe, Kayode Philip Fadahunsi, Simon Smith, Griphin Baxter Chirambo, Emma Larsson, Patrick Henn, Mala Mawkin, John O’ Donoghue

**Affiliations:** 1 School of Medicine University College Cork Cork Ireland; 2 Department of Primary Care and Public Health Imperial College London London United Kingdom; 3 Faculty of Health Sciences Mzuzu University Mzuzu Malawi; 4 Ashford and St Peter's Hospitals NHS Trust Chertsey United Kingdom; 5 Assert Centre College of Medicine & Health University College Cork Cork Ireland; 6 School of Medicine Imperial College London London United Kingdom; 7 Malawi eHealth Research Centre University College Cork College Road Cork Ireland

**Keywords:** mHealth app, health app, mobile health, health website, quality, quality assessment, methodology, high-income country, low-income country, middle-income country, LMIC, mobile phone

## Abstract

**Background:**

In recent years, there has been rapid growth in the availability and use of mobile health (mHealth) apps around the world. A consensus regarding an accepted standard to assess the quality of such apps has yet to be reached. A factor that exacerbates the challenge of mHealth app quality assessment is variations in the interpretation of quality and its subdimensions. Consequently, it has become increasingly difficult for health care professionals worldwide to distinguish apps of high quality from those of lower quality. This exposes both patients and health care professionals to unnecessary risks. Despite progress, limited understanding of the contributions of researchers in low- and middle-income countries (LMICs) exists on this topic. Furthermore, the applicability of quality assessment methodologies in LMIC settings remains relatively unexplored.

**Objective:**

This rapid review aims to identify current methodologies in the literature to assess the quality of mHealth apps, understand what aspects of quality these methodologies address, determine what input has been made by authors from LMICs, and examine the applicability of such methodologies in LMICs.

**Methods:**

This review was registered with PROSPERO (International Prospective Register of Systematic Reviews). A search of PubMed, EMBASE, Web of Science, and Scopus was performed for papers related to mHealth app quality assessment methodologies, which were published in English between 2005 and 2020. By taking a rapid review approach, a thematic and descriptive analysis of the papers was performed.

**Results:**

Electronic database searches identified 841 papers. After the screening process, 52 papers remained for inclusion. Of the 52 papers, 5 (10%) proposed novel methodologies that could be used to evaluate mHealth apps of diverse medical areas of interest, 8 (15%) proposed methodologies that could be used to assess apps concerned with a specific medical focus, and 39 (75%) used methodologies developed by other published authors to evaluate the quality of various groups of mHealth apps. The authors in 6% (3/52) of papers were solely affiliated to institutes in LMICs. A further 15% (8/52) of papers had at least one coauthor affiliated to an institute in an LMIC.

**Conclusions:**

Quality assessment of mHealth apps is complex in nature and at times subjective. Despite growing research on this topic, to date, an all-encompassing appropriate means for evaluating the quality of mHealth apps does not exist. There has been engagement with authors affiliated to institutes across LMICs; however, limited consideration of current generic methodologies for application in LMIC settings has been identified.

**Trial Registration:**

PROSPERO CRD42020205149; https://www.crd.york.ac.uk/prospero/display_record.php?RecordID=205149

## Introduction

### Background

Mobile health (mHealth) apps can be defined as software “incorporated into smartphones to improve health outcome, health research, and health care services” [1]. In 2017, >325,000 mHealth apps were available for download [2]. These apps can enhance health promotion and disease prevention, resulting in improved patient outcomes and economic savings [3,4].

In 2020, 35% of US health care consumers used mHealth apps compared with just 16% in 2014 [5]. Access to and use of these apps is also increasing in many low- and middle-income countries (LMICs) [6]. In 2015, there were >7 billion mobile telephone subscriptions worldwide, 70% of which were in LMICs [7,8]. Furthermore, 95% of the global population resides in an area covered by mobile cellular networks, with 84% of people having access to mobile broadband networks [9]. Such widespread use and access to smartphones has helped incorporate mHealth solutions into health care systems within LMICs [10].

Since the introduction of mHealth in the late 2000s, apps have facilitated improvements in disease management, reductions in health care costs and boosted service efficiency [3,4,11]. Despite the growing popularity of mHealth apps, research has also identified the potential risks associated with their use. Regardless of location, quality of content and software functionality are areas of concern in mHealth apps [12], as are data privacy and security [10,13]. For successful implementation of mHealth in LMIC settings, additional factors such as user-prospective and technical factors should also be considered [10].

At present, there is no comprehensive, universally available methodology to assess the quality of mHealth apps [14]. In addition, the existing five-star rating scales available within app stores provide subjective indications of quality, which are often unreliable [15]. Given the paucity of current methodologies, unreliability associated with star ratings, and the ever-expanding mHealth app market, the challenge for health care professionals to identify high-quality apps is becoming increasingly difficult.

A factor that exacerbates the conundrum of quality assessment is indeed the word quality itself. Quality can be considered an umbrella term encompassing many dimensions, depending on its context. Hence, disparities exist in the depth and focus of its definition. The Institute of Medicine defines quality in health care broadly as “the degree to which health services for individuals and populations increase the likelihood of desired health outcomes and are consistent with current professional knowledge” [16]. The International Organization for Standardization adopts a more expansive approach and defines quality as the “degree to which a set of inherent characteristics of an object fulfills requirements” [17]. Nouri et al [18] proposed a broad classification model to address quality in relation to the mHealth app evaluation. Within this model, criteria and subcriteria are outlined for consideration when evaluating the quality of mHealth apps.

Perhaps it is this hierarchical, multifaceted nomenclature that has rendered it difficult to unify on a standard of quality when discussing mHealth apps. Various approaches have been taken to help identify higher quality apps. In the United Kingdom, the National Health Service app library provides a collection of mHealth apps of approved quality [19]. The Federal Institute for Drugs and Medical devices in Germany is set to examine the quality of apps with a view to doctors ultimately being able to “prescribe health care apps to patients” [20].

Efforts are also being made for mHealth app evaluation methods in LMIC settings [21]. However, despite the rapidly increasing market access, significant developments have yet to occur. Given the variability of socioeconomics across the globe, additional parameters in methodologies for mHealth app evaluation in emerging economics may be required.

### Objectives

The primary aim of this rapid review is to identify current methodologies in the literature to assess the quality of mHealth apps. Second, it aims to determine what aspects of quality these methodologies consider. Third, it aims to examine global research input on this topic since 2005. Finally, this review examines the applicability of such methodologies in LMIC settings. 

## Methods

### Study Design

Rapid reviews draw upon traditional systematic review processes to accelerate and streamline research while preserving the rigor and quality of review methodology [22]. Given the aforementioned research aims and objectives, a rapid review approach was deemed appropriate.

The broad principles of scoping review methodology, as defined by Arksey and O’Malley [23], were followed to formulate the research question and identify relevant studies for selection. A concept-centric approach was taken for the charting procedure in line with the advice given by Webster and Watson [24] for writing literature reviews in the field of information sciences. A standard protocol was followed in accordance with the PRISMA-ScR (Preferred Reporting Items for Systematic Reviews and Meta-Analyses extension for Scoping Reviews) checklist [25].

### Search Strategy

A systematic search strategy was developed and applied across four databases: PubMed, EMBASE, Scopus, and Web of Science. These databases have strong scientific and medical focuses. This combination of databases was chosen in an effort to guarantee adequate and efficient coverage of relevant papers [26].

Under the guidance of an academic librarian and reflecting upon the advice of Arksey and O’Malley [23] for conducting literature reviews, the research question was split into the following four specific concepts: *methodology, assess, quality,* and *mHealth app*. Through the iterative process of keyword searching and preliminary search testing using Medical Subject Headings terms, synonyms of each concept were incorporated into the search string. The final search string is provided in Multimedia Appendix 1. An intrinsic link exists between mHealth apps and health websites. Therefore, variations of *health website* were included in the search string to identify papers that potentially covered both mHealth app and health website domains.

The search was conducted in December 2020 and was limited to studies published in English between 2005 and 2020. The year 2005 was chosen as the starting point for this review as the first iPhone was released on the market in 2007, and the app store was created in 2008 [27]. Geographical restrictions were not imposed on this search.

### Study Selection

**Figure 1:**
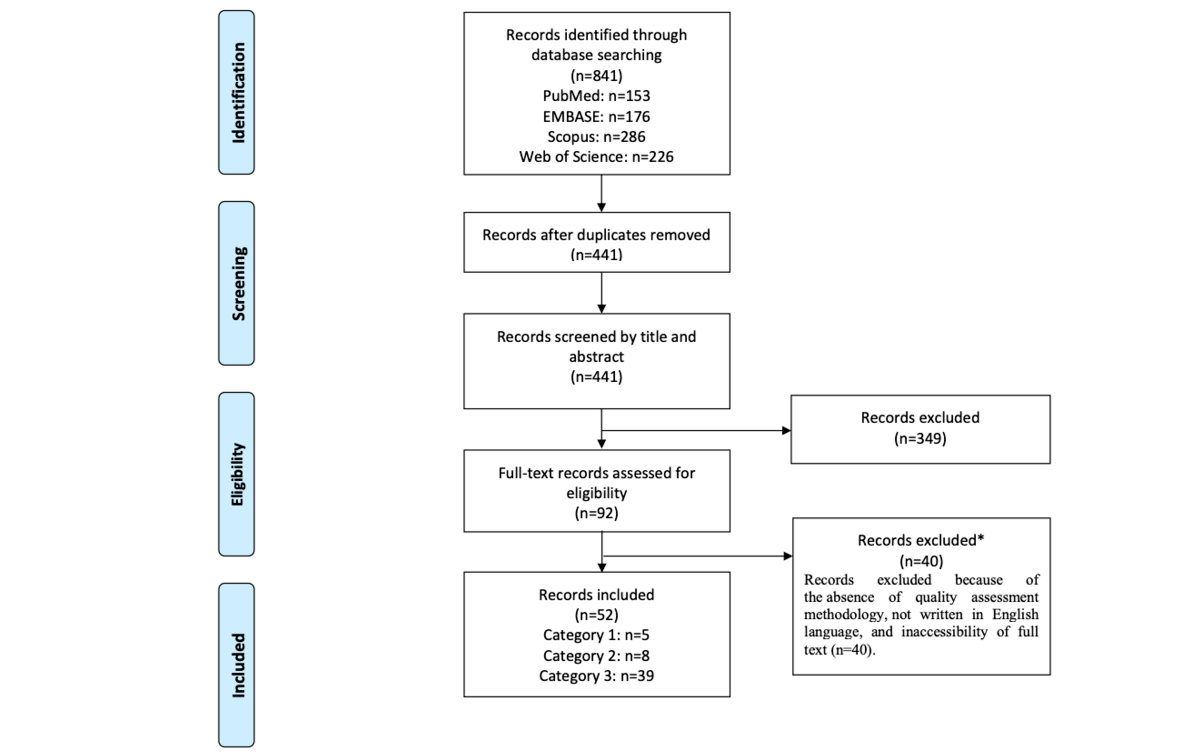
PRISMA (Preferred Reporting Items for Systematic Reviews and Meta-Analyses) flow diagram of the inclusion process.

The reference management software EndNote X9 (Clarivate Analytics) was used to collate the initial literature search citations. Duplicates were removed before exporting the remaining citations to the Covidence systematic review software (v2409). The author FW initially screened titles and abstracts to determine whether a paper met the general study selection criteria. The full texts of the remaining papers were formally screened against the inclusion and exclusion criteria by FW. Any papers that the author FW was unsure about were screened by and clarified through engagement with the author JOD. The search results were presented in a PRISMA flow diagram (Figure 1).

### Selection Criteria

The inclusion criteria for studies were as follows:

1. Papers that proposed a methodology to evaluate the quality of mHealth apps (regardless of assessing one aspect or several aspects of quality)
2. Papers that used a methodology for the quality evaluation of specific groups of mHealth apps
3. Papers published in English from 2005 onward in research journals

The exclusion criteria for studies were as follows:

1. Papers that proposed a methodology for the evaluation of non-mHealth apps
2. Papers that proposed a methodology solely for the evaluation of health websites
3. Papers that were not available through the library of University College Cork, interlibrary loans, or via direct communication with authors
4. Papers that were not available in the English language

### Categorization of Reviewed Papers

Papers that successfully passed full-text screening were subdivided into three categories based on their thematic synergies. These were as follows:

- Category 1 (generic methodologies): papers that proposed generic methodologies to evaluate the quality of mHealth apps or mHealth websites
- Category 2 (health condition–specific methodologies): papers that proposed methodologies designed specifically to evaluate the quality of mHealth apps that focus on one medical condition (ie, App Quality Evaluation Tool to evaluate the quality of nutrition apps)
- Category 3 (use of existing methodologies): papers that used a prepublished methodology to evaluate certain groups of mHealth apps

### Data Extraction

Data items extracted from all studies included paper title, author, year, aim or objective, name of methodology used or developed, target platform of methodology, disease focus of study, strengths, validity and reliability of methodology, weaknesses, and future work. The location of authors institute affiliations was classified based on the World Bank Classification system [28] into high-, middle-, or low-income countries. Any uncertainty was clarified through discussion with a second reviewer (JOD). This extraction form was initially piloted and amended where necessary.

The methodologies proposed by the papers in category 1 (ie, those that proposed a generic methodology for mHealth app evaluation) were compared with a reference classification checklist of criteria for assessing mHealth app quality proposed by Nouri et al [18].

### Quality Evaluation

The aim of this rapid review is to assess the extent of published literature on mHealth quality assessment methodologies and related studies rather than to evaluate specific causes and effects. Therefore, as supported by the World Health Organization, risk of bias assessment was not conducted, as this review served as an information gathering process [29].

## Results

The search and paper retrieval processes are illustrated in Figure 1. A total of 841 potentially relevant papers were identified. Of the 841 papers, following the removal of duplicates, 441 (52.4%) papers remained, with 52 (6.2%) papers meeting the criteria for inclusion.

### Characteristics of Retrieved Papers

The papers were subdivided into three categories. Category characteristics and respective citations are indicated in Table 1.

**Table 1:** Summary of paper categories and their respective citations (N=52).

Category	Number of papers, n (%)	General explanation of category
Category 1: generic methodologies	5 (10)	Papers that propose generic methodologies to evaluate the quality of mHealth apps and health websites [30] Papers that propose generic methodologies to evaluate the quality of mHealth apps [31-34]
Category 2: health condition–specific methodologies	8 (15)	Papers that propose methodologies designed specifically to evaluate the quality of mHealth apps which focus on one medical condition (ie, AQEL^a^ to evaluate the quality of nutrition apps) [35-42]
Category 3: use of existing methodologies	39 (75)	Papers that use a prepublished methodology to evaluate certain groups of mHealth apps (ie, the use of MARS^b^ to evaluate the quality of traditional medicine mHealth apps available in Iran) [43-81]

^a^AQEL: App Quality Evaluation Tool.

^b^MARS: Mobile App Rating Scale.

### Category 1

Of the five papers identified in category 1, 1 (20%) proposed a methodology to evaluate the quality of both mHealth apps and health websites [30], and 4 (80%) proposed methodologies solely used to evaluate the quality of mHealth apps [31-34]. The coverage of the Nouri et al [18] mHealth app evaluation criteria found in the methodologies within this category can be viewed in Table 2.

**Table 2:** Coverage of category 1 methodologies of the criteria for assessing the quality of mHealth apps proposed by Nouri et al [18].

mHealth app quality assessment criteria (Nouri et al [18])		Baumel et al (Enlight) [30]	Yasini et al [31]	Anderson et al (ACDC^a^) [32]	Stoyanov et al (MARS^b^) [33]	Martínez-Pérez et al [34]
**Design**						
	Suitability of design	✓				
	Aesthetics	✓			✓	
	Appearance	✓				✓
	Design consistency	✓		✓	✓	
**Information or content**						
	Credibility	✓	✓	✓	✓	✓
	Accuracy	✓	✓		✓	✓
	Quality of information	✓		✓	✓	
	Quantity of information	✓		✓	✓	
**Usability**						
	Ease of use	✓	✓	✓	✓	✓
	Operability		✓			
	Visibility	✓				
	User control and freedom					
	Consistency and standards					
	Error prevention					✓
	Completeness	✓	✓			
	Information needs	✓	✓			
	Flexibility and customizability		✓			
	Competency					
	Style	✓				
	Behavior	✓				
	Structure					
**Functionality**						
	Performance			✓	✓	✓
	Health warnings			✓		
	Feedback	✓		✓		
	Connectivity and interpretability		✓	✓		
	Record	✓				
	Display					
	Guide	✓				
	Remind or alert	✓				
	Communicate	✓				✓
**Ethical issues**						
	Beneficence	✓	✓			
	Nonmaleficence		✓			
	Autonomy		✓			
	Justice		✓			
	Legal obligations	✓	✓			
**Security and privacy**						
	Security	✓	✓			✓
	Privacy	✓	✓			
**User perceived value**						
	User perceived value	✓	✓	✓	✓	✓

^a^ACDC: App Chronic Disease Checklist.

^b^MARS: Mobile App Rating Scale.

### Category 2

The methodologies proposed in category 2 of the papers focused specifically on asthma [35], pain management [36], medication adherence [37], medication-related problems [38], hard of hearing [39], diabetes mellitus [40], infant feeding [41], and nutritional [42] mHealth apps. The methodologies proposed within this category of papers were highly specific to one topic of medicine. Therefore, their respective dimensions of quality were not subjected to further investigation.

### Category 3

Papers in category 3 were concerned with a variety of medical conditions. Quality assessment of nutrition-related mHealth apps was the most prevalent area of research within this category [46,47,50,55,58,63,65,76]. Other types of mHealth apps studied were those related to obesity and weight management [53,62,64], pain management [43,70,75], mental health [51,67], and oncology [57,82].

Within this category, of the 39 papers, 23 (59%) papers applied one methodology, and 16 (41%) papers used a combination of methodologies to evaluate the quality of mHealth apps. The Mobile App Rating Scale (MARS) construct [33] was the most commonly used methodology (24/39, 62%). It was used in 38% (15/39) of papers as the sole means of quality evaluation [45-49,52,57,69-71,73-76,78,79]. MARS was also used in an additional 23% of papers along with other methodologies, such as clinical guidelines [46,55,56,58,65,67,68,77,81]. The breakdown of methodologies and the frequency of their use can be viewed in Table 3.

**Table 3:** Breakdown of methodologies used by papers in category 3 (N=39).

Methodology used	Articles, n (%)
MARS^a^ [45-49,52,57,69-71,73-76,78,79]	15 (38)
uMARS^b^ [48,63]	2 (5)
Silberg scale [51,53,64]	3 (8)
AQEL^c^ [50]	1 (3)
mHON^d^ code [61]	1 (3)
IOM^e^ aims [72]	1 (3)
Combination of methodologies (System Usability Scale and clinical guidelines) [46,47,54-56,58-60,62,65-68,77,80,81]	16 (41)

^a^MARS: Mobile App Rating Scale.

^b^uMARS: user version of Mobile App Rating Scale.

^c^AQEL: App Quality Evaluation Tool.

^d^mHON: Mobile applications–Health on the Net.

^e^IOM: Institute of Medicine.

### Timeline of Published mHealth Assessment Methodologies and Studies

**Figure 2:**
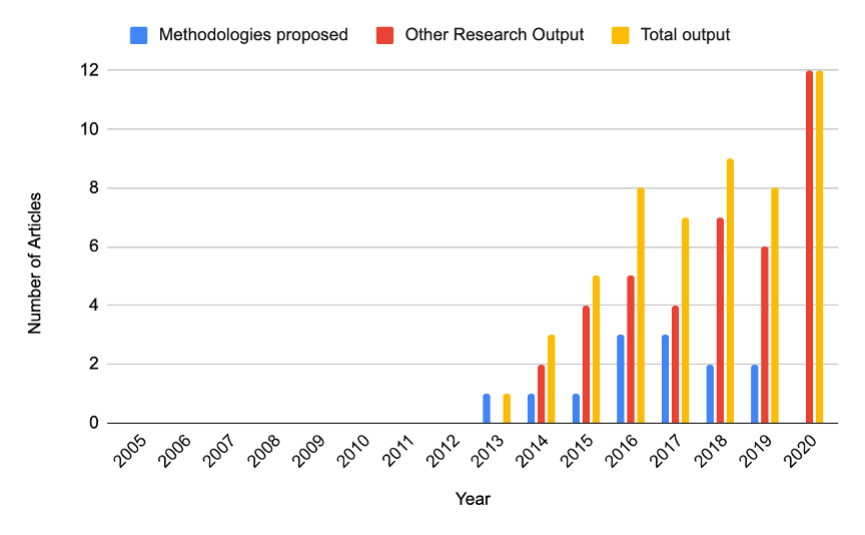
Illustration of research output on mobile health app evaluation studies from 2005 to 2020.

Research output on the topic of mHealth quality assessment has significantly increased in recent years. Since 2005, 52 papers have been published on this topic; of the 52 papers, 12 (23%) were published in 2020 alone. The research output of novel methodologies for evaluating mHealth apps (categories 1 and 2) and studies relating to the topic (category 3) since 2005 are illustrated in Figure 2.

### International Input

**Figure 3:**
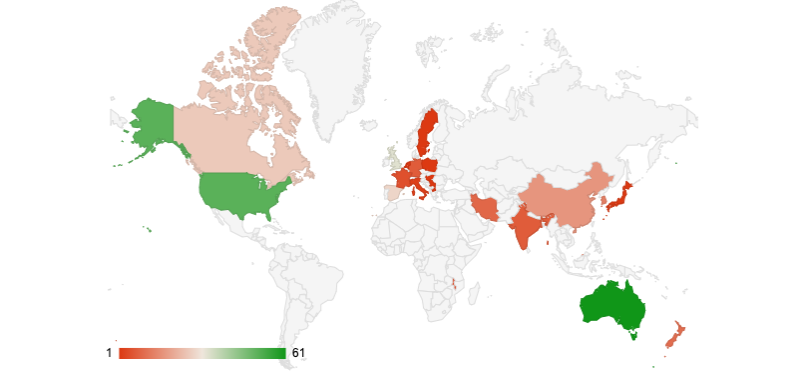
Geo chart indicating research output affiliated to each country.

The location of authors institute affiliations for all papers (categories 1, 2, and 3) was classified into high-, middle-, or low-income countries based on the World Bank Classification system [28]. All category 1 papers were published by authors affiliated with institutions in high-income countries. One paper in category 2 was published by authors affiliated with an institute in a low-or middle-income country [35]. Of the 39 papers in category 3, 2 (5%) were solely published by authors affiliated with institutes in LMICs [44,78]. A further 21% (8/39) of papers in this category had at least one author affiliated with institutes in LMICs [45,54,56,60,63,67,74,75]. The location of authors’ affiliated institutes can be viewed in the Geo chart in Figure 3. A breakdown of countries and the number of authors affiliated with it can be viewed in Table 4.

**Table 4:** A breakdown of research contribution based on the country of author affiliation institute.

Author affiliation and country (ordered alphabetically)		Number of authors affiliated to institutions in that country, n (%)
**High-income countries**		
	Australia	61 (23.7)
	Canada	26 (10.1)
	France	5 (1.9)
	Germany	7 (2.7)
	Hungary	1 (0.4)
	Italy	2 (0.8)
	Japan	1 (0.4)
	Korea, Republic	13 (5.1)
	Netherlands	1 (0.4)
	New Zealand	10 (3.9)
	Poland	1 (0.4)
	Spain	28 (10.9)
	Singapore	16 (6.2)
	Sweden	1 (0.4)
	United Kingdom	33 (12.8)
	United States	51 (19.8)
Total affiliations from high-income countries		257 (100)
**Middle-income countries**		
	China	17 (48.6)
	India	7 (20)
	Iran, Islamic Republic	9 (25.7)
	Macedonia	1 (2.9)
	Serbia	1 (2.9)
Total affiliations from middle-income countries		35 (100)
**Low-income countries**		
	Brunei	3 (42.9)
	Malawi	4 (57)
Total affiliations from low-income countries		7 (100)

## Discussion

### Principal Findings

A variety of methodologies to assess the quality of mHealth apps have been identified in this review. Some adopted a generic approach and can be used to evaluate mHealth apps for various medical conditions. Other methodologies take a disease-centric approach and are only relevant when considering apps concerned with that particular disease. Despite a number of quality assessment methodologies being available, significant variations in the dimensions of quality that they address were identified. Given the subjective nature of quality and its subdimensions, it is not surprising to find this high degree of diversity.

As presented in category 3, the MARS construct proposed by Stoyanov et al [33] has been widely used by other authors to evaluate the quality of mHealth apps. MARS is a concise, easy-to-use tool that covers many of the Nouri et al [28] criteria for assessing mHealth app quality (Table 2). Despite its popularity, MARS fails to address some important key aspects of quality, most notably security and privacy. The use of mHealth apps may involve the processing of sensitive information by multiple parties. Therefore, a rising awareness and concern exist in relation to the safety of the information that they contain [13,83]. This underscores the importance of considering privacy and security when evaluating mHealth apps and highlights a significant limitation of the MARS construct. Only two of the five generic methodologies took both of these dimensions into consideration [30,31].

In contrast to MARS, the Enlight suite of assessments proposed by Baumel et al [30] provides a more thorough assessment of quality. It has been designed for both mHealth apps and health website quality evaluation purposes. As presented in Table 2, Enlight has comprehensive coverage of the Nouri et al [28] criteria for mHealth app quality assessment. Rating measures within the Enlight suite are divided into two sections: quality assessments and checklists. The quality assessment section refers to aspects of quality that relate to the user’s experience of an mHealth app. The checklists are not expected to directly impact the end user’s experience of the product’s efficacy; rather, these lists may expose the user (or provider) to acknowledged risks or benefits.

Respondent fatigue is a well-documented phenomenon in questionnaires [84]. Although the Enlight suite provides a far-reaching means to evaluate the quality of mHealth apps, its all-encompassing nature may, in reality, curtail its use. Along with the checklists section, 28 questions are contained within the Enlight Quality Assessment section. This is significantly greater than that of other generic methodologies. Hence, the use of the Enlight suite would take significantly longer than others to score the quality of mHealth apps. Undeniably, a greater balance is needed to maximize user uptake and engagement among the health care community. This is especially important, as in many cases health care professionals are not allotted additional time to assess new apps.

Although an abundance of mHealth apps is available, academic studies on their clinical impact are lacking. Concerningly, many mHealth apps are not based on any behavior change theory, and in many cases, their effectiveness has not been correctly evaluated [82,85]. With that said, the ability of apps to stimulate behavior change is becoming a growing area of interest [86]. The behavior change technique is not an explicit quality criterion proposed by Nouri et al [28]; however, the World Health Organization recognizes the importance of health outcome–based measures [87]. This review identified its considerations in three of the generic methodologies [30,32,33]. The App Chronic Disease Checklist (ACDC) construct includes behavior change as a singular point of consideration [32]. In contrast, the MARS construct assesses “the perceived impact of an app on the user’s knowledge, attitudes, intentions to change as well as the likelihood of actual change in the targeted health behavior” in its *App-Specific* section [33]. Similarly, the *Enlight Suites Therapeutic Persuasiveness* section is specifically dedicated to addressing the topic of behavior change techniques [30]. Although behavior change technique in itself is a broad concept, it is reassuring to identify its consideration even to a certain extent within many methodologies.

### Challenges in App Assessment

As mentioned in the introduction, a paucity of uniform definitions for quality and its respective subdimensions exists. A lack of clear-cut definitions not only poses a challenge to this research but also adds a level of ambiguity to mHealth app quality evaluations as a whole. Until precise definitions of each dimension of quality are provided, ongoing subjectivity regarding the interpretation of a dimension of quality with respect to an mHealth app may continue.

It is quite important to consider the validity and reliability of the assessment tools in health care [88]. Validity indicates how well a tool measures what it intends to measure, and reliability expresses the extent to which the obtained results are reproducible [88]. Most of the selected tools offered some form of face and content validity based on expert opinions [30-33]. Only 4% (2/52) of studies [30,33] provided reliability results. However, the selected studies did not conduct factor analysis, which can limit their construct validity. In addition, none of the tools provided any predictive validity, which is the extent to which the scores predict the ability of the mHealth app to improve the targeted health condition. Thus, the paucity of information on the validity and reliability of the available tools could limit their usefulness in practice.

Methodologies proposed within category 1 provide the user with a means to assess the quality of mHealth apps. However, no methodology within this category provides the user with a scoring mechanism or rubric to interpret the results. For example, when using the ACDC checklist, what does it mean if an app contains an overwhelming amount of information but scores perfect results in all other dimensions of quality? Does this render the app low quality? A lack of clear scoring mechanisms may hinder a user’s interpretation of the evaluation process, making it an inconclusive exercise.

### Applicability of mHealth App Evaluation Methodologies in High-, Middle-, and Low-Income Countries

Although 46% of new mHealth app publishers are from Europe [89], the apps they develop are often available in international markets. As the functionality of mHealth apps becomes more diverse and ownership of smartphones rises, it is likely that their adoption by those living in LMICs will continue to increase. The applicability of the aforementioned methodologies for assessing mHealth app quality in LMIC settings has not been widely considered. As a health care professional contemplates whether a specific mHealth app would be beneficial for their patient, the suitability of an app in the context of his or her patient must be considered. Various regulatory, technical, and user-prospective factors have been identified as obstacles to the integration of mHealth solutions in resource-poor settings [10].

Many regulatory factors that may affect mHealth use in LMICs also affect their use in high-income countries (HICs). Security and privacy of data are two examples. Table 2 highlights that these factors are currently considered in many quality assessment methodologies. Continued access to the internet represents a technological factor that may affect mHealth use in LMICs disproportionately to that in HICs [10]. Despite the penetration rate of mobile broadband signal doubling in LMICs over the past two decades [90], challenges such as use, cost, and speed continue to exist. As such, researchers may wish to consider the impact of inconsistent internet services on an app's functionality. The ACDC checklist [32] was the sole generic methodology to address the facilitation of an *offline mode*. The incorporation of questions such as this within methodologies helps to consider the reality faced by many within LMICs at present.

Socioeconomics can impact the use of mHealth solutions. With increased global demand, it represents an important parameter for consideration. Two factors within the domain of socioeconomics, which may be important, are cultural appropriateness and literacy. Cultural appropriateness is essential for designing user interfaces or web interfaces for international and country-specific audiences that will be accepted and liked by users [91]. Cultural appropriateness applies to mHealth app evaluations not only in LMICs but also in HICs. If the content of an mHealth app is unsuitable for a particular audience, its download may become a contentious or fruitless exercise. For example, an app designed for prenatal care in Ireland may not be appropriate for use in sub-Saharan Africa. As far as the authors are aware, no generic methodology has explicitly examined the cultural appropriateness of an mHealth app. However, vague considerations were made in the MARS construct [33] and the Enlight suite [30]. In these cases, the suitability of certain aspects of an app, such as information and visual content with respect to the target audience, were mentioned. Given the broadening cultural diversity of app users, perhaps a more formal effort to consider cultural appropriateness exists for the benefit of those in LMICs and HICs.

Health literacy is a concern for many low- and middle-income populations. Within the domain of literacy, readability refers to the comprehension level required by an individual to correctly understand and engage with written material [92]. Past research indicates that many mHealth apps are written at excessively high reading grade levels [66,93]. Poor readability may increase the scope for misinterpretation and render an app inaccessible to many potential end users [66,93]. Nouri et al [28] considered readability as a subcriterion of ease of use [18]. Only two of the five generic methodologies explicitly consider these subcriteria [31,32]. Although the average reading level in LMICs is rising, in many cases, it is still behind that of HICs [94]. Given the proportion of mHealth app development from HICs, a salient need for health care professionals in LMICs to consider the readability of these apps in terms of their potential end users is important.

### Future Work

The authors identify several directions for future work in this area of research. First, the review could be extended to papers published in languages other than English, providing a more accurate representation of quality assessment methodologies currently available at an international level.

The Enlight suite provides a thorough means for evaluating the quality of mHealth apps; however, its fundamental usability and ability to consider an app in the context of various populations could be enhanced. An area of active research by the authors is the revision and enhancement of this tool based on the knowledge of this rapid review. Through a Delphi study and supporting survey techniques, the suite is in the process of being modified to make it more user friendly and comprehensive in LMIC settings.

This study highlights several challenges associated with the use of quality assessment methodologies in practice. There is scope to formalize methodology reliability processes, yielding more transparency and comparability in assessments. A scoring mechanism or rubric may be considered in future methodologies that provides users with a means to summarize an app based on the aggregated dimensions of quality that it fulfills.

On a practical level, this research provides additional emphasis on the importance of mHealth app quality assessments. Methodologies such as the Enlight suite and MARS construct are suitable for the purposes outlined in this paper. However, going forward, these methodologies may also be used in consultation with health care professionals for reasons of app development, providing a template for quality assurance.

### Strengths

This review has several strengths. To the best of the authors’ knowledge, this is the first review to consider the applicability of generic methodologies to evaluate the quality of mHealth apps in LMIC settings. Furthermore, it highlights the affiliations of authors institutes, indicating where significant research input has come from in the past. This review begins to consider further parameters that one may wish to incorporate into methodologies in the future to improve their relevancy across resource-poor settings.

### Limitations

This research is not without limitations. A decision was made by the research team to exclude methodologies for the evaluation of *health websites only* and non-mHealth apps. This decision was based on the fact that such methodologies often consider parameters that are not applicable to mHealth apps themselves. In an effort to retrieve all relevant papers, terms relating to these concepts were included within the search string. However, only those papers that formally met the inclusion or exclusion criteria were considered in this review.

Although reviewed by a second author where necessary, paper retrieval, selection, and data extraction were completed by one reviewer (FW). Nouri et al provided generalized definitions or examples of its respective quality assessment criteria [18]. A considered approach was taken by the author FW, whereby a methodology with reasonable coverage of the criteria was positively reflected in data extraction. A lack of universal definitions for quality and its respective subdimensions posed a challenging factor for data extraction, comparison, and synthesis on this topic.

The investigators acknowledge that the country affiliation of methodology authors may have limited relevance toward the application of those methodologies within their respective locations. Nevertheless, given the international market demand and varying socioeconomics, the investigators believe that this approach serves as one of many, which may help indicate the suitability of mHealth app quality assessment methodologies in LMICs.

Finally, only articles published in English were included in this review. This may have some impact on our results presented in Figure 3 and Table 4, as methodologies published in other languages were not identified.

### Conclusions

Quality assessment of mHealth apps is a complex task. Significant heterogeneity exists between the aspects of quality that are considered by the methodologies identified by this rapid review. Some key aspects of quality remain unaddressed by certain methodologies despite their growing popularity. Although engagement with authors affiliated to institutes in LMIC exists on this topic, limited consideration has been made for the use of current methodologies in LMIC settings.

Owing to the variety of stakeholders involved in mHealth (eg, software engineers, information technology departments or companies, health care professionals, and patients), the challenges of finding or developing an all-encompassing methodology to assist health care professionals in assessing the quality of a given app is easily appreciated. With the ever-increasing role of mHealth apps in health care, it is time to consider policy development at the international level. An inclusive and intuitive mHealth app assessment methodology is required to ensure the reliable use of mHealth apps worldwide.

## Appendix

Multimedia Appendix 1 Search string applied to databases.

## Abbreviations

ACDC: App Chronic Disease Checklist
HIC: high-income country
LMIC: low- and middle-income country
MARS: Mobile App Rating Scale
mHealth: mobile health
PRISMA-ScR: Preferred Reporting Items for Systematic Reviews and Meta-Analyses extension for Scoping Reviews
PROSPERO: International Prospective Register of Systematic Reviews
