# Supplementary material for: Identification and Evaluation of Methodologies to Assess the Quality of Mobile Health Apps in High-, Low-, and Middle-Income Countries: Rapid Review
Source: JMIR Mhealth Uhealth. 2021 Oct 12;9(10):e28384. doi: 10.2196/28384 (PMC8548973; doi:10.2196/28384)
Supplement: Multimedia Appendix 1 [file mhealth_v9i10e28384_app1.docx]

### Appendix 1

Search String

|  | Concept | Concept terms |
| --- | --- | --- |
|  |  |  |
| #1 | Methodology | TI=(Framework OR System OR Tool* OR Model OR Algorithm OR Questionnaire OR Survey OR Scheme OR Process OR Checklist OR Protocol OR Approach OR Method* OR Scor* OR “Rating Scale” OR Scale OR Instrument OR Checklist OR Principle OR Rubric) |
| #2 | Assess | TI=(Assess* OR Evaluat* OR Appraise OR Determine OR Check OR Gauge OR Estimate OR Validat* OR Analysis OR Rate OR Screen OR Judge) |
| #3 | Quality | TI=(“Data Quality” OR DQ OR “Information Quality” OR IQ OR “Fit-for-purpose” OR “Fit-for-use”) |
| #4 | mHealth app | TI=(mHealth OR “mHealth App*” OR “Mobile Health” OR mHealth OR “Health Mobile” OR App* OR “Smartphone App*” OR “Medical App*” OR “eHealth App*” OR “eHealth mobile App” OR “Electronic Health” OR “Digital Health” |
|  |  | OR “Ubiquitous Health” OR uHealth OR Telemedicine OR “Health IT” OR “mHealth Platform” OR “mHealth Program” OR “Mobile eHealth Interventions”OR “Clinical App” OR “Health Website” OR “Health Web Site”) |
| Search string: |  | (#1 OR #2) AND (#3) AND (#4) |
